# Sentinel lymph node biopsy in early stage cervical cancer: A meta‐analysis

**DOI:** 10.1002/cam4.3645

**Published:** 2020-12-13

**Authors:** Xinyue Zhang, Bingting Bao, Sixue Wang, Mingyu Yi, Li Jiang, Xiaoling Fang

**Affiliations:** ^1^ Department of Gynecology and Obstetrics The Second Xiangya Hospital Central South University Changsha Hunan P.R. China

**Keywords:** blue dye, cervical cancer, indocyanine green, sentinel lymph node, technetium, ultrastaging

## Abstract

**Background:**

The aim of this study was to determine the specific side detection rate of the sentinel lymph node biopsy and the accuracy in predicting lymph node metastasis in early stage cervical cancer.

**Methods:**

A systematic search of databases was performed from the inception of the databases to 27 June 2020. Studies of cervical cancer patients with FIGO stage FIGO ⅠA~ⅡB, evaluating the sentinel lymph node biopsy with blue dye, technetium 99, combined technique (blue dye with technetium 99) or indocyanine green with a reference standard of systematic pelvis lymph node dissection or clinical follow‐up were included. Stata12.0 and Meta‐Disc 1.4 were used for the meta‐analysis.

**Results:**

Of 2825 articles found, 21 studies (2234 women) were eventually included. Out of 21 studies, 20 met the detection rate evaluation criteria and six were included for sensitivity meta‐analysis. Due to heterogeneity, it was inappropriate to pool all studies. The pooled specific side detection rates were 85% in tumors up to 2 cm, 67% in tumors over 2 cm, 75.2% for blue dye, 74.7% for technetium 99, 84% for combined technique, and 85.5% for indocyanine green. The sentinel lymph node biopsy had a pooled specific side sensitivity of 88%. Adverse effects of sentinel lymph node biopsy appear minimal for most patients and are mainly related to the injection of blue dye.

**Conclusions:**

Sentinel lymph node biopsy using a tracer with a high detection rate and ultrastaging is highly accurate and reliable when limited to seriously selected patients, with satisfactory bilateral lymph node mapping and where enough cases for learning curve optimization exist. Indocyanine green sentinel lymph node mapping seems to be a superior sentinel lymph node mapping technique compared to other methods at present.

## Graphical abstract

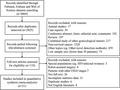

## 1 INTRODUCTION

Cervical cancer is the third most common cancer in women, worldwide,^1^ and is presently afflicting younger women. With the recent development of early diagnosis and treatment, the detection rate of early cervical cancer has increased significantly. Lymph node metastasis is an independent risk factor in the prognosis of cervical cancer. In 2018, the International Federation of Gynecology and Obstetrics included the lymph node status in their updated staging classification of cervical cancer.^2^ Currently, the standard surgical procedure for early cervical cancer is radical hysterectomy plus bilateral pelvic lymphadenectomy. However, postoperative pathological statistics show that only 15%‐20% of early cervical cancer patients actually had lymph node metastasis, meaning that more than 80% without lymph node metastasis underwent unnecessary pelvic lymph node dissection.^3^ The procedure is difficult, time‐consuming, costly, and has many potential postoperative complications, including vascular and nerve injury, lymphatic cyst, lower extremity lymphedema, and lymphatic leakage. Accurate preoperative and intraoperative assessment of the status of the pelvic lymph nodes is essential for the proper selection of the optimum early cervical cancer treatment.

As the first stop for tumor lymphatic drainage, the sentinel lymph node (SLN) reflects the status of the corresponding regional lymph nodes. The SLNs can be identified during surgery by lymphoscintigraphy using technetium 99 (^99m^Tc), blue dye, indocyanine green (ICG) and so on, and examined histopathologically using frozen section, hematoxylin and eosin (H&E) or ultrastaging with serial sectioning and immunohistochemistry (IHC) using the anticytokeratin antibody. Sentinel lymph node biopsy (SLNB) has been applied to the prediction of lymph node metastasis of penile,^4^ vulvar^5^ and breast cancer.^6^ The aim of this study was to systematically review and perform a meta‐analysis of the role of SLNs in early cervical cancer. As the cervix is a midline structure and lymphatic drainage is bilateral, we try to assess the lymph nodes on each side separately. This study 1) assessed the specific side detection rate (SSDR) of the SLNB using different tracer methods for different sized tumors, 2) determined the accuracy of the SLNB in predicting lymph node metastasis in early cervical cancer, 3) summarized the false negative cases, localizations of the SLNs in early cervical cancer and the adverse events related to the SLNB reported in the literature.

## 2 MATERIALS AND METHODS

### 2.1 Search strategy, selection criteria and data abstraction

**Figure 1:**
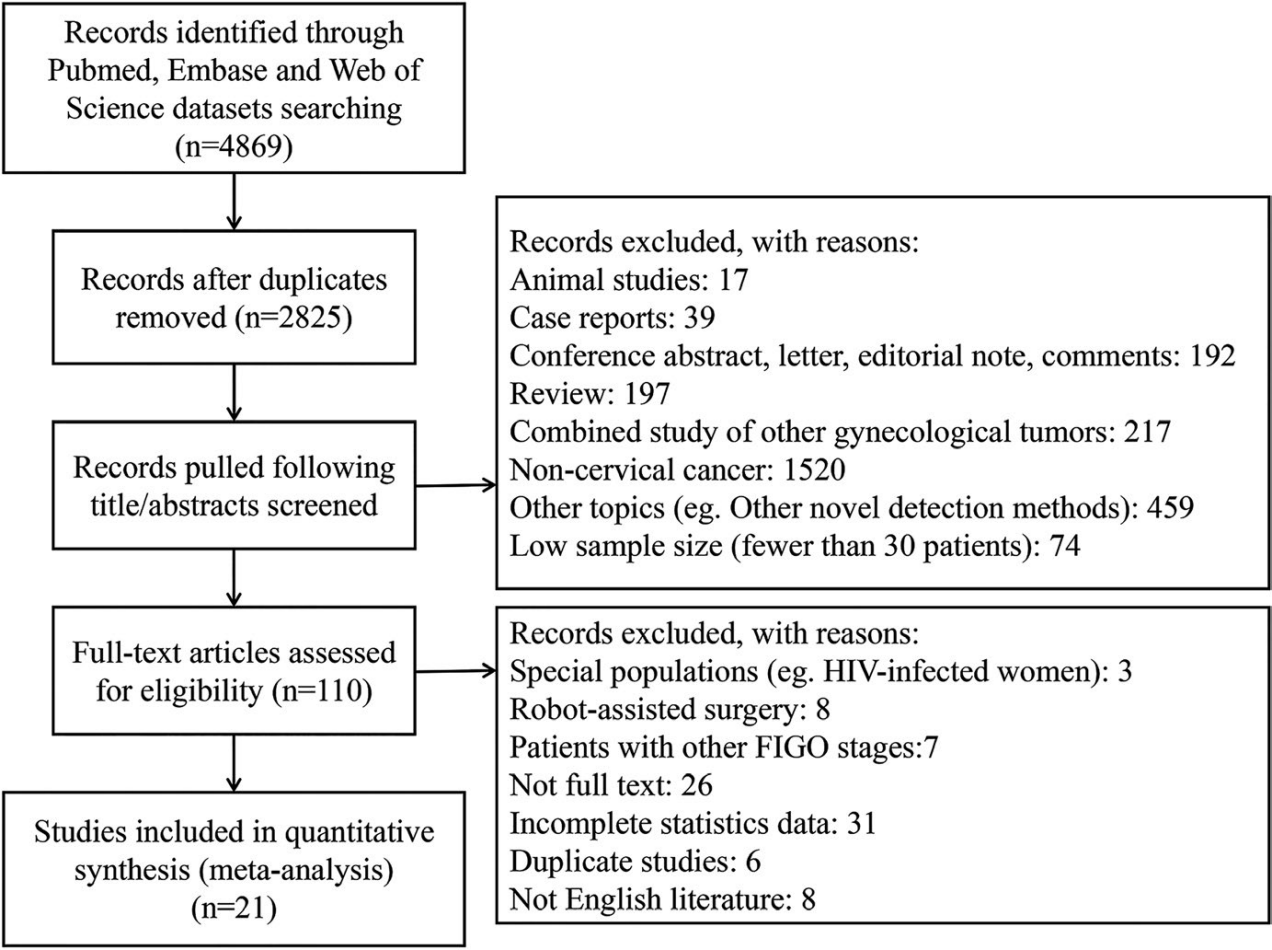
The PRISMA diagram for diagnostic review.

The study subjects were cervical cancer patients (FIGO ⅠA~ⅡB) who underwent an SLNB. Three databases, PubMed, Embase, and Web of Science, were systematically searched for relevant references from the inception of the databases to 27 June 2020. We used a broad inclusive search strategy so as not to miss a seminal contribution. Both MeSH terms and text words were used, which included “sentinel lymph node” and “cervical cancer”. The specific search strategy could be obtained from the author. Endnote software was used to manually filter out duplicate articles. The literature search process is shown in Figure 1.

Inclusion criteria: 1) Official publication in English language, 2) Patients with cervical cancer (FIGO ⅠA~ⅡB, in which patients in IA1 had the complication of lymph vascular space invasion), 3) Sample size ≥30 cases (experienced learning curve),^7^ 4) Laparotomy or laparoscopic surgery, 5) The methods were SLNB using blue dye,^99m^Tc, combined technique (blue dye with^99m^Tc) or ICG. If multiple detection methods were used simultaneously in an article, the subgroup with sample size≥30 cases was included in the study.

Evaluation rationale: In order for studies to be included for the detection rate evaluation, we needed to be able to extract the total number of hemi‐pelvis and the number of hemi‐pelvis with detected SLNs during the operation, or calculate the SSDR from the article data. Studies for sensitivity evaluation included those in which the gold standard was systematic pelvic lymph node dissection or clinical follow‐up for SLN‐negative patients, and which reported the number of hemi‐pelvis with pelvic lymph node involvement (identified by systematic pelvic lymph node dissection or pelvic recurrence during follow‐up) and the number of false‐negative hemi‐pelvis, or the specific side sensitivity (SSS) could be calculated from the article data.

Exclusion criteria: 1) Repetitive publications, 2) No data required or incomplete statistics data, 3) Not full‐text, 4) Combined study of other gynecological tumors, 5)Robot‐assisted surgery.

### 2.2 Data extraction and quality evaluation

Two investigators screened the literature independently and extracted and checked the data, and a third investigator resolved discrepancies. Articles published by the same author or agency were discussed to identify possible duplicate studies, if any, including recently published studies. The extracted data mainly included: the first author, year published, sample size, FIGO staging, number of pelvic sidewalls, tracer, SSDR, histopathological techniques (the presence of ultrastaging, multiple slices, serial sections, step sections or additional sections in an article were considered “ultrastaging”), true positive (TP), true negative (TN), and false negative (FN). In order to obtain more complete data, we tried to contact the corresponding authors if necessary. We assessed the quality of the included studies with the Quality Assessment of Diagnostic Accuracy Studies checklist (QUADAS‐2)^8^ using Revman 5.3.

### 2.3 Statistical analysis

The SSDR, SSS, TP, TN, FN, and FP of each study were obtained from the source articles, or calculated based on the data provided when the information could not be extracted directly. Since the presence of a positive SLN indicated lymph node metastasis, there was no false positive result in this study. The positive predictive value and specificity were always 1.0. SSDRs and their 95% confidence intervals were calculated by Stata 12.0 (Stata, College Station, TX, USA). The diagnostic meta‐analysis was performed by Meta‐Disc 1.4 (Unit of Clinical Biostatistics team of the Ramón y Cajal Hospital, Madrid, Spain). On the basis of investigation of heterogeneity, summary estimates of sensitivity, negative likelihood ratios (the ratio of the FN rate to the TN rate), and the diagnostic odds ratio (the ratio of the positive likelihood ratio to the negative likelihood ratio) were derived as appropriate. The *I* ^2^ statistic and the Cochrane Q test were used to quantify the extent of the heterogeneity. If heterogeneity was low (*I* ^2^<50% and *p* > 0.1), a fixed effect model was used to calculate the effect size, otherwise, the random effect model was used. Results were graphically displayed with forest and receiver operating curve plots. The publication bias was assessed by funnel plots using Stata 12.0.

## 3 RESULTS

### 3.1 Summary of study characteristics

A total of 4869 articles were retrieved through electronic search, of which 110 articles were evaluated in full‐text and 21 relevant studies were included. Figure 1 shows the PRISMA diagram. Out of the 21 studies, 20 met the criteria of detection rate evaluation and six were included for sensitivity meta‐analysis. Five of the articles contained different subgroups, in which each subgroup used different detection strategies, and each subgroup separately met the inclusion criteria. We regarded each subgroup as a separate study. The characteristics of the included studies are summarized in Table 1.

**Table 1:** The characteristic of the included studies

Number	Reference	Year	Cases	No. of pelvic sidewalls	Stage	Tracer	Histopathological techniques	Detection rate (100%)	True positive (TP)	False positive (FP)	False negative (FN)	True negative (TN)	Sensitivity (100%)
1	Dargent^11^	2000	35	69	IA2‐IB2	Dye	H&E	85.50%	11	0	0	48	100%
2	Levenback^9^	2002	39	78	IA1‐IIA	Dye+Tc	H&E; ultrastaging	98.70%					
3	Rob1^30^	2005	100	200	IA2–IIA	Dye	H&E; ultrastaging	71%					
4	Rob2^30^	2005	83	166	IA2–IIA	Dye+Tc	H&E; ultrastaging	93.37%					
5	Stefano^10^	2005	50	100	IA2–IIA	Dye	H&E; ultrastaging	72%	11	0	1	60	91.67%
6	Angioli^31^	2005	37	74	IB1	Tc	H&E; ultrastaging	50.50%					
7	Silva^25^	2005	56	104	IA2–IIA	Tc	H&E; ultrastaging	71%					
8	Wydra^18^	2006	100	200	IB1–IIA	Dye+Tc	H&E; ultrastaging	75%					
9	Bats^19^	2008	68	136	IA2‐IIB	Dye+Tc	H&E; ultrastaging	66.18%					
10	Pažin^15^	2008	50	100	IB‐IIA	Dye	NR	65%					
11	Ogawa^12^	2010	82	164	IA–IIB	Tc	H&E	76.83%	15	0	0	111	100%
12	Cormier1^16^	2011			IA1‐IIA	Dye	H&E; ultrastaging		10	0	1	79	90.90%
13	Cormier2^16^	2011			IA1‐IIA	Dye+Tc	H&E; ultrastaging		15	0	1	99	98.70%
14	Diaz^17^	2011	63	126	IA1‐IIA	Dye+Tc	H&E; ultrastaging	84.92%					
15	Roy1^22^	2011	152	304	IA1‐IIA	Dye	H&E; ultrastaging	80.92%					
16	Roy2^22^	2011	166	332	IA1‐IIA	Tc	H&E; ultrastaging	91.87%					
17	Xuelian^13^	2011	68	136	IA2‐IB1	Tc	IHC	67.65%					
18	Martínez^21^	2013	94	188	IA–IB1	Dye+Tc	H&E; ultrastaging	90.43%					
19	Freitas^32^	2014	57	114	IA2–IIA	Dye+Tc	H&E; ultrastaging	65.80%	9	0	1	65	90%
20	Bats^33^	2015	139	278	IA1–IB1	Dye+Tc	H&E; ultrastaging	86.33%					
21	Beavis^34^	2016	30	60	IA1–IB2	ICG	H&E; ultrastaging	93.30%					
22	Buda1^20^	2016	76	152	IA2–IB1	Dye+Tc	H&E; ultrastaging	86.15%					
23	Buda2^20^	2016	68	136	IA2–IB1	ICG	H&E; ultrastaging	99.25%					
24	Tanaka1^14^	2016	106	212	IA‐IIB	Tc	H&E	85.80%	19	0	8	155	70.30%
25	Tanaka2^14^	2016	43	86	IA‐IIB	ICG	H&E	61.60%	4	0	1	48	80%
26	Dostálek^35^	2018	350	700	pT1a‐pT2	Dye+Tc	H&E; ultrastaging	86.50%					

Abbreviations: Dye: blue dye; H&E: hematoxylin and eosin staining; ICG: indocyanine green; NR: not recorded; Tc: technetium 99.

### 3.2 Quality evaluation of the included studies

**Figure 2:**
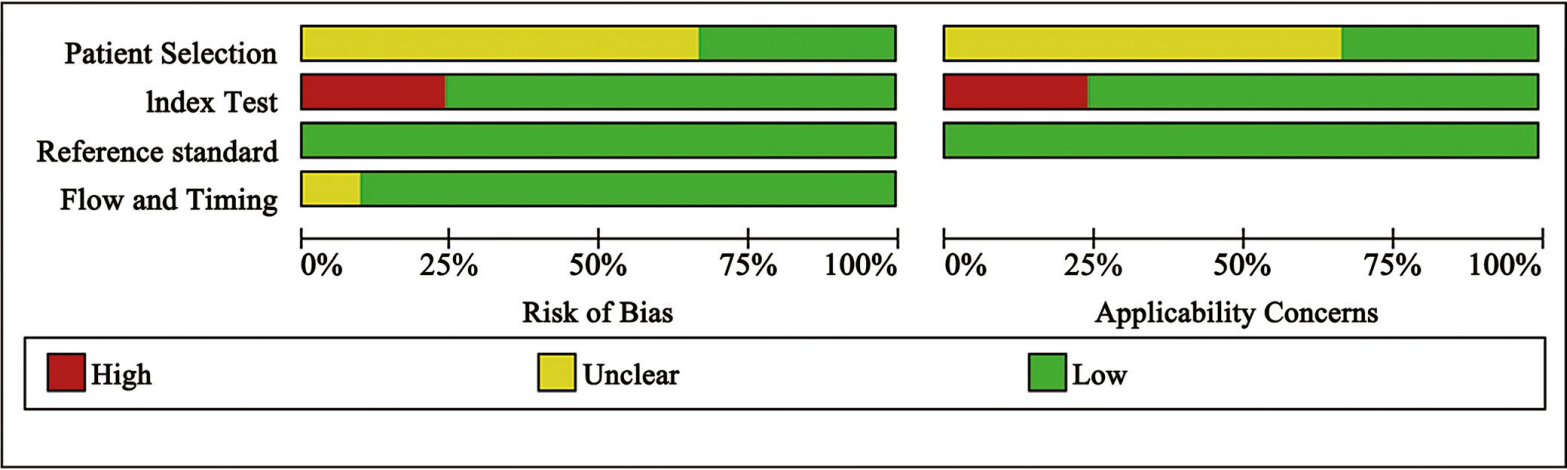
Quality assessment of the included studies

We evaluated the quality of the included studies with the QUADAS‐2 checklist (Figure 2). Among the 21 included studies, 14 indicated only the time frame for inclusion of cases but did not indicate whether they were consecutive or random cases. Two studies did not perform histological examination on all SLNs.^9^, ^10^ The combination of H&E and ultrastaging was considered the best histopathology examination strategy, but four studies only used H&E or IHC,^11^, ^12^, ^13^, ^14^ and one study did not mention the strategy used,^15^ which might lead to a high risk of bias and affect the evaluation of the diagnostic accuracy.

### 3.3 Specific side detection rate of the SLNB in early cervical cancer patients

**Figure 3:**
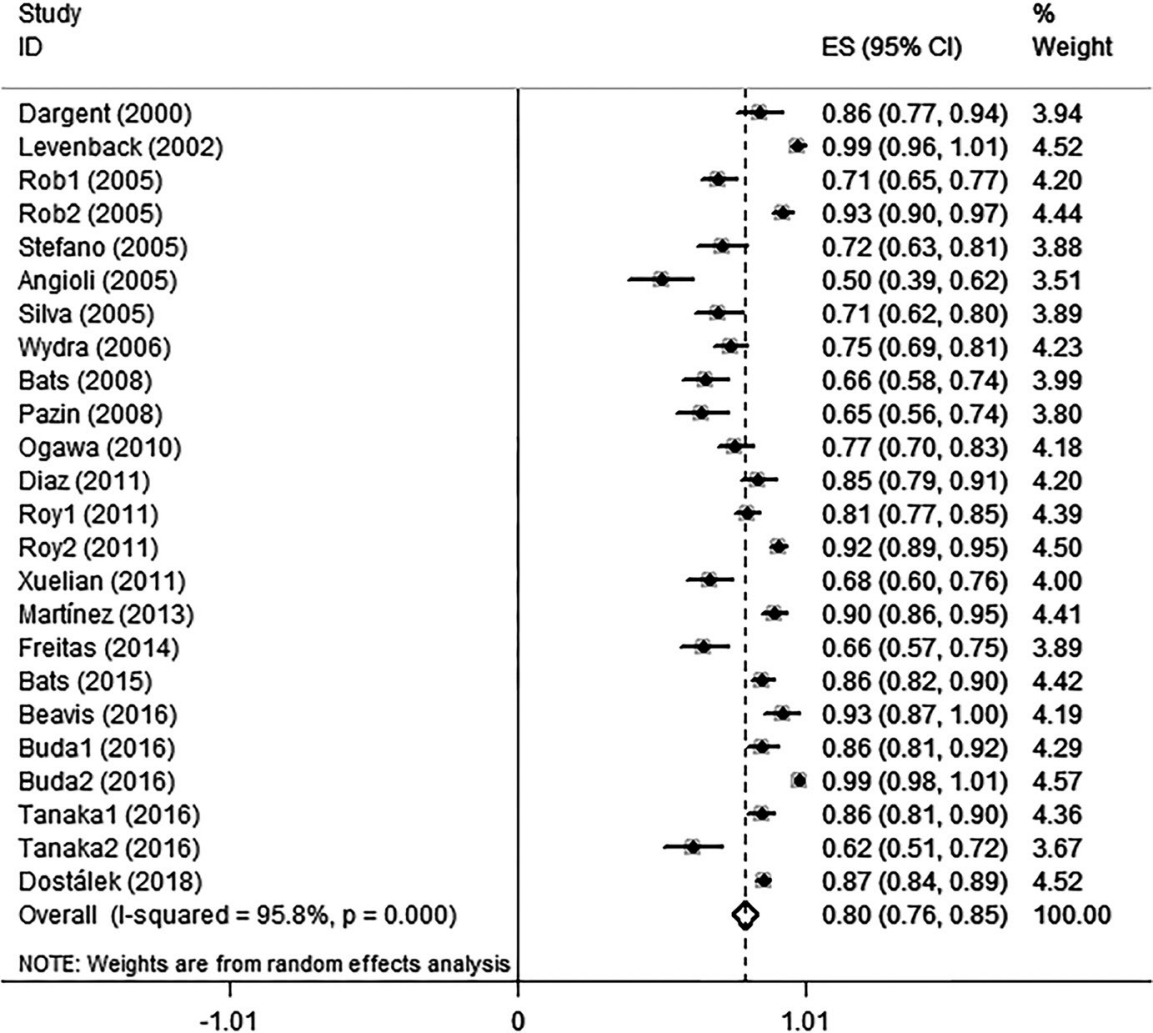
Forest plot of SSDR of SLNB

**Figure 4:**
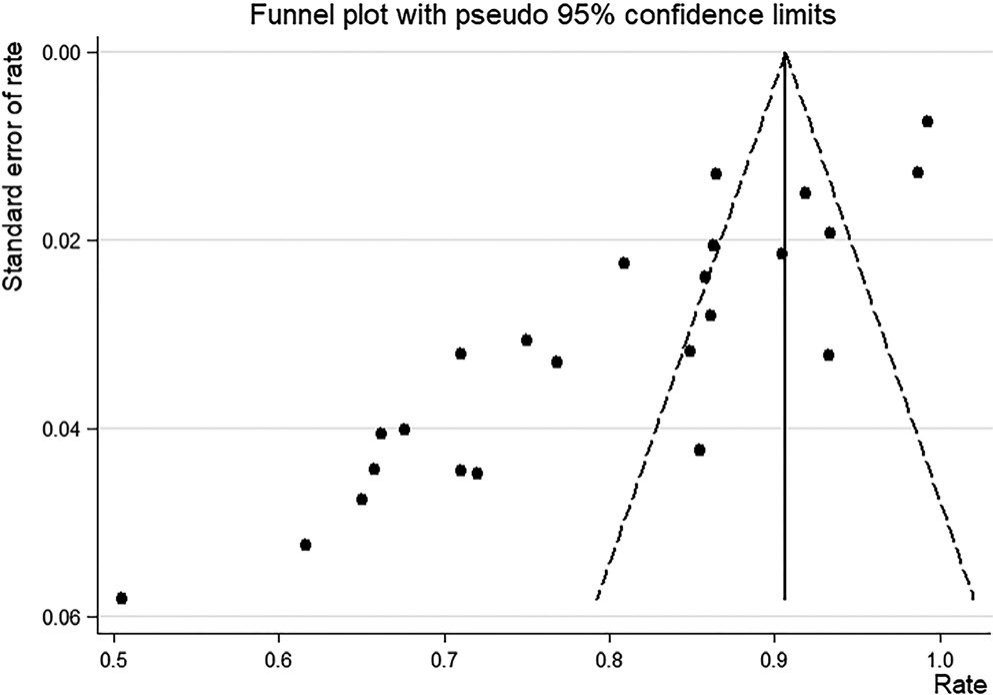
Funnel plot of SSDR of SLNB

Regarding each subgroup that met the inclusion criteria as an independent study, a total of 24 studies (2112 patients) were included in the assessment of the detection rate. The tracers used for the SLNB included blue dye,^99m^Tc, combined technique (blue dye with^99m^Tc) and ICG. The pooled SSDR of the four methods was 80% (95%CI: 76%‐85%).The forest plot is shown in Figure 3. *I* ^2^=95.8% (*p* = 0.000), which indicated a high degree of heterogeneity. Accordingly, we performed a subgroup analysis. The subgroup analysis of studies with tumors ≤2 cm vs >2 cm showed a pooled SSDR of 85% (95%CI: 80%‐90%) vs 67% (95%CI: 53%‐80%), respectively. The subgroup analysis of studies using blue dye,^99m^Tc, or the combined technique (blue dye with^99m^Tc) vs ICG for the SLNB showed a pooled SSDR of 75.2% (95%CI: 68.5%‐82%), 74.7% (95%CI: 64.4%‐85%), and 84% (95%CI: 78.5%‐89.5%), respectively, vs 85.5% (95%CI: 68.9%‐1.022%). Among these, the pooled SSDR of ICG was the highest. Other types of subgroups were not analyzed here because the relevant data could not be obtained. The funnel plot shown in Figure 4 suggested the existence of publication bias, butt Duval‐Tweedie's trim and fill method showed that no trimming was performed.

### 3.4 Accuracy of the SLNB in early cervical cancer

**Figure 5:**
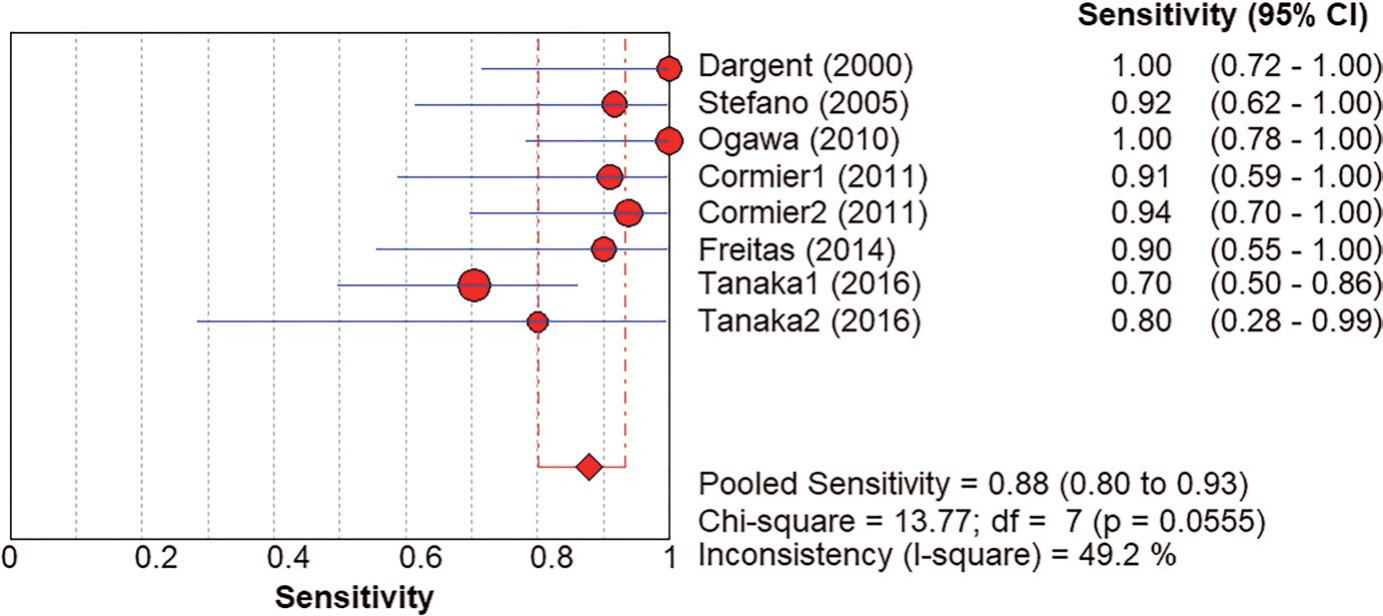
Forest plot of SSS of SLNB

**Figure 6:**
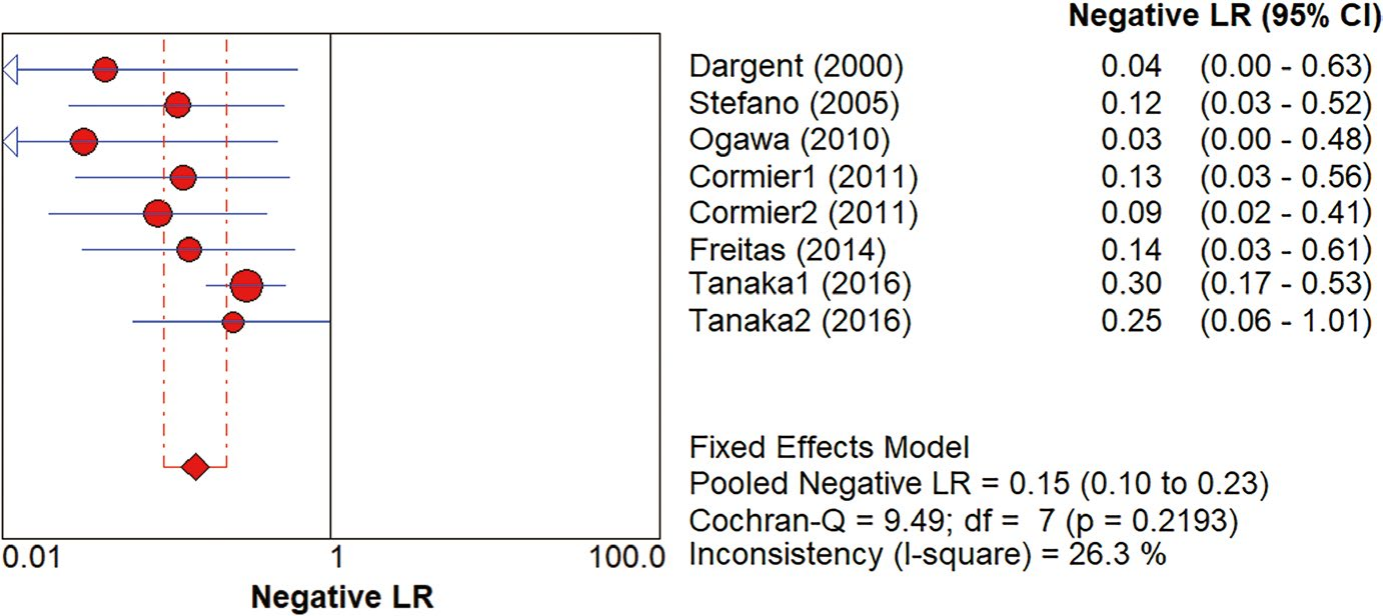
The negative likelihood ratios of SLNB

**Figure 7:**
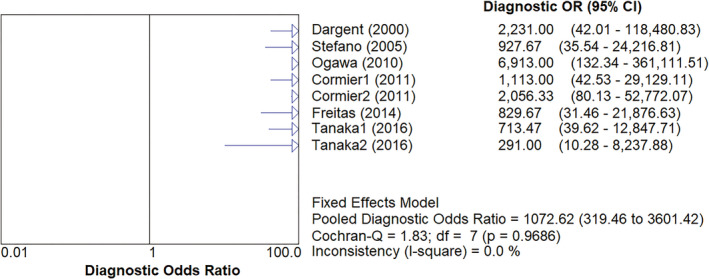
The pooled diagnostic odds ratio of SLNB

**Figure 8:**
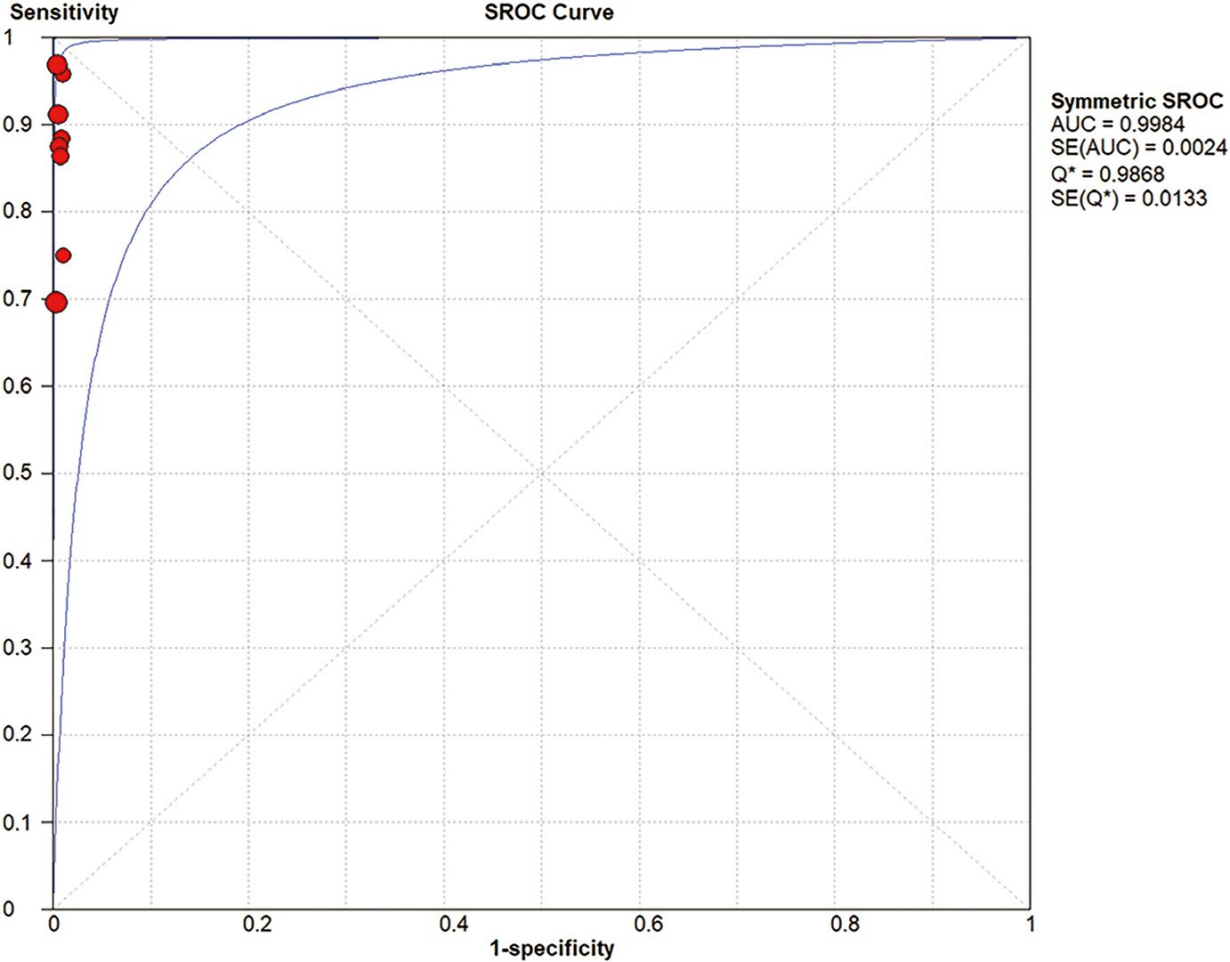
The receiver operating characteristic curve of SLNB

**Figure 9:**
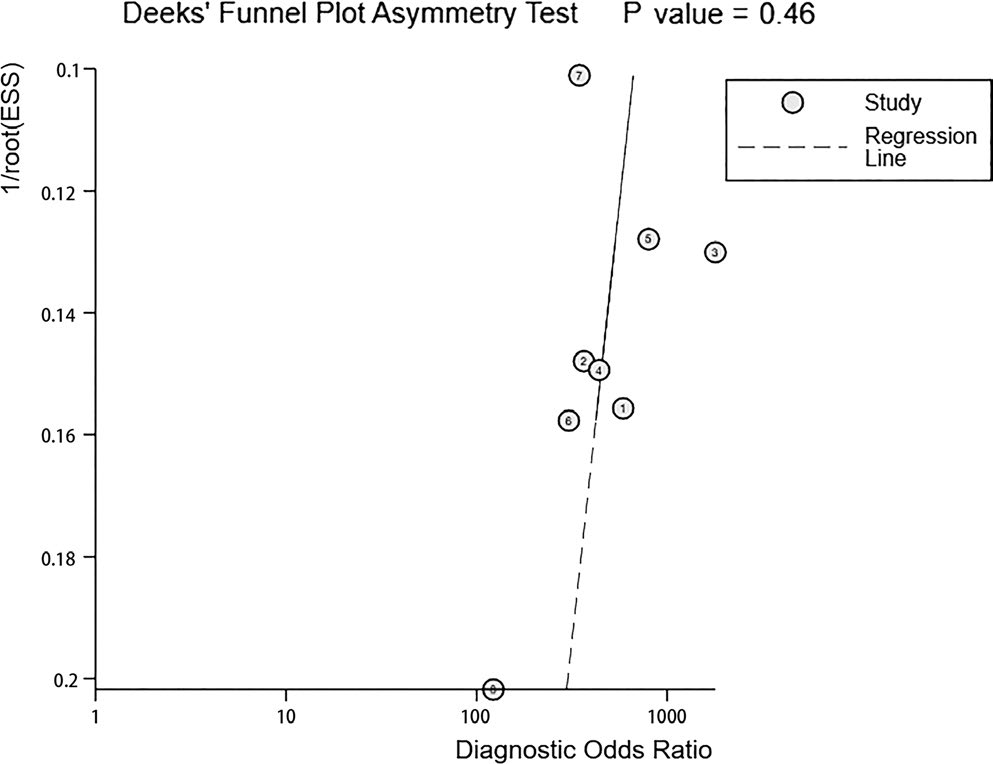
Deek's Funnel plot of SSS of SLNB

Regarding each subgroup that met the inclusion criteria as an independent study, eight studies were included in the assessment of the accuracy of the SLNB diagnosis. The forest plot of the sensitivity meta‐analysis is shown in Figure 5. The pooled SSS was 88% (95%CI: 80%‐93%), *I* ^2^=49.2% (*p* = 0.0555). The subgroup analysis of studies using only H&E staining vs ultrastaging showed a pooled SSS of 84% (95%CI: 73%‐93%) vs 92% (95%CI: 80%‐98%). The pooled negative likelihood ratio was 16% (95%CI: 9%‐29%), as shown in Figures 6, *I* ^2^=26.3% (*p* = 0.2193). The pooled diagnostic odds ratio was 1072.62 (95%CI: 319.46–3601.42), as shown in Figures 7, *I* ^2^=0.0% (*p* = 0.9686). The area under the receiver operating characteristic curve was 0.9984 (Figure 8). Figure 9 shows the Deeks’ funnel plot for the diagnostic meta‐analysis (*p* = 0.46 > 0.05). The symmetry detected in the funnel plot indicated low publication bias in these statistically significant models. However, the number of original studies included in this analysis is small, so the test efficacy of the funnel plot is low.

### 3.5 Description of the FN cases

Of those FN cases in the included studies, on final pathology, most had both negative SLN and positive non‐SLN parametrial lymph nodes in the same basin.^10^, ^16^, ^17^ Parametrial lymph nodes, especially in the medial part, located near the cervix, where the radioactivity was high and the blue coloration was intense, were particularly difficult to identify.^9^, ^10^ Wydra et al.^18^ reported that in all FN cases, the primary cervical tumor was >2 cm, and there was an isthmus infiltration. Tanaka et al.^14^ demonstrated that the FN rate of patients with tumor diameter ≥2 cm was higher than that of patients with tumor diameter <2 cm (8.6% vs 1.4%, *p* < 0.01). Bats et al.^19^ reported that two FN cases happened in patients with locally advanced stages of cervical cancer (stage IIB). Buda et al.^20^ reported that the only FN case was recruited in a center that had just started the experience with SLNB, where the learning curve was not yet well established.

### 3.6 Localizations of SLNs in early cervical cancer

The most common localizations of SLNs were the external iliac, the internal iliac, the interiliac, the hypogastric, the obturator, and the bifurcation areas. Some SLNs were found in unconventional drainage areas, such as the common iliac, the para‐aortic, the presacral, the cardinal ligament, and the parametrial regions. The percentage of SLNs in different abnormal areas varied widely from article to article. Levenback et al.^9^ reported that 9% of SLNs were found in the para‐aortic and parametrial regions, and 11.4% were found in the common iliac area. Martínez et al.^21^ showed that SLNs were located in the common iliac area in 11.1% of cases, in the presacral area in 1.5% of cases, and in the infra‐mesenteric para‐aortic area in 0.8% of cases. Roy et al.^22^ reported that 3.8% of SLNs were found in the para‐aortic region, including two with metastasis. Other studies also found more or less SLNs in abnormal areas.

### 3.7 Adverse events related to SLNB

Only two articles mentioned adverse events related to SLNB. Levenback et al.^9^ reported that tolerance for the whole process was excellent, overall. No allergic reaction occurred during the injection of isosulfan blue or^99m^Tc radiocolloid. A few patients experienced a transient decrease of oxygen saturation. Some patients reported the^99m^Tc radionuclide injection was painful but the feeling was very brief (one or two minutes). Plante et al.^23^ reported that anaphylaxis occurred in two patients secondary to the injection of blue dye. One patient developed blue hives in the abdomen that disappeared within a few hours. Another patient suffered profound vasomotor shock a few minutes after the injection of blue dye which needed aggressive resuscitation and the operation was cancelled. A week later, she made a full recovery, was injected with^99m^Tc only, and underwent the planned surgery, uneventfully. In addition, one patient developed transient spurious pulse oximetry desaturation due to the injection of blue dye. Finally, most patients had green or blue colored urine, usually lasting 24–48 hours.

## 4 DISCUSSION

In today's society, it is advocated to avoid excessive medical treatment, to reduce the scope of surgery while ensuring the surgical effectiveness, and to pay attention to the quality of life after surgery, so that patients can benefit to the maximum extent. Thus, the most important role of an accurate SLNB technique would be the identification of node‐negative patients with cervical cancer in whom an unnecessary systematic pelvic lymph node dissection could be avoided.

According to O'Boyle et al.,^24^ due to the bilateral drainage of the cervical lymphatics, SLN mapping ideally requires the identification of at least one SLN in each hemi‐pelvis, otherwise it is “technically inadequate”. As some studies^25^ reported that some cases had intense unilateral parametrial infiltration and the SLN could not be identified in the same basin. While the positive non‐SLN lymph nodes were just in the basin of the parametrial involvement, suggesting that the lymphatic drainage was blocked by the parametria. Thus, if only "patient‐specific" analysis is carried out, the meaning is very limited. Therefore, regarding each hemi‐pelvis as a distinct unit, we performed a “side‐specific” analysis. However, through the full‐text analysis of the selected articles, we found that “side‐specific” analysis was not carried out in most of the studies. Here we strongly urge future researchers to carry out “side‐specific” analysis.

The pooled SSDR was 80% (95% CI: 76%‐85%), and the heterogeneity test showed *I* ^2^ = 95.8% (*p* = 0.000). To investigate the heterogeneity, we performed subgroup analysis focusing on tumor size and tracer type. The results showed that the pooled SSDR of the studies with tumors ≤2 cm was 18% more than the studies with tumors>2 cm, which suggested that patients with smaller tumor size might be more suitable for SLNB. Possible reasons for tumor size affecting the SLN detection rate: 1) The larger the tumor, the less the normal cervical structure, which makes the injection of the tracer more difficult and affects the development rate. 2) For larger tumors, the probability of lymph node metastasis increases. Tumor cells block lymphatic vessels, leading to a high possibility of tracer diversion, thus affecting the detection rate of truly metastatic lymph nodes. The pooled SSDR of the studies using ICG as tracer was 1.5% more than the studies using the combined technique, and it was much higher than using blue dye or^99m^Tc alone. There are several advantages over the traditional blue dye/^99m^Tc mapping technique other than the potential to improve the detection rate. It protects patients and staff from radiation exposure and does not require radiology staff. In addition, ICG is injected when the patient is anesthetized, avoiding the pain of preoperative radiocolloid injection. Lastly, the cost of ICG is significantly lower than that of the combined technique because no additional injection time and lymphoscintigraphy are required.^26^ To sum up, ICG seems to be a superior tracer for SLN mapping compared to other methods, indicating that further studies on ICG and other fluorescent dyes is worthwhile. In this meta‐analysis, the sensitivity of SLNB for pelvic lymph node staging is the point we are more interested in. The pooled SSS was 88% (95%CI: 80%‐93%), which was also fairly high. The pooled negative likelihood ratio was 16% (95%CI: 9%‐29%), indicating that when the result of SLNB is negative, the possibility of true negative is very high. The pooled diagnostic odds ratio was 1072.62 (95%CI: 319.46–3601.42), suggesting that the discriminant effect of the diagnostic method was remarkable. Moreover, the summary receiver operating characteristic curve showed good symmetry, indicating that there was no obvious threshold effect. AUC = 0.9984, suggesting that the diagnostic accuracy of SLNB was very high. Our analysis confirmed a good diagnostic performance of SLNB for assessing lymph node metastasis in early cervical cancer. The results of further subgroup analysis showed that the pooled SSS of the studies using only H&E staining was 8% less than those using ultrastaging. That is because ultrastaging can reveal isolated tumor cells and tumor micrometastasis less than 2 mm which is difficult to detect by routine pathology.^17^ It can improve the detection rate of lymph node metastasis, especially micrometastasis.^27^ Therefore, ultrastaging should be performed for all SLNs with negative H&E staining to detect the tumor micrometastasis and determine the choice of subsequent treatment.^28^, ^29^

By summarizing the FN cases in the included studies, on final pathologic evaluation of hysterectomy specimens, we found that special attention should be paid to the parametrial lymph nodes, especially those in the medial part, due to the characteristics difficult to detect by SLN mapping. In addition, the localization of SLNs has given rise to some new thinking due to the finding that direct lymphatic drainage outside the conventional dissection field to unusual drainage areas may be a reason for lymph node recurrence after negative complete pelvic lymph node dissection. Some SLN development areas are not involved in routine pelvic lymphadenectomy for early cervical cancer (such as para‐aortic and presacral lymph nodes), or are easily missed in postoperative pathological examinations (such as para‐uterine lymph nodes). However, these lymph nodes appear as the SLNs of cervical cancer, and some even appear as the only SLN, suggesting that routine lymph node dissection within the same areas for all patients may lead to missing some important first‐stop lymph nodes, or even those that have already metastasized. Therefore, SLN mapping in early stage cervical cancer seems to be very important. Adverse effects of the SLNB technique appear minimal for most patients and are mainly related to the injection of blue dye. However, the risk of serious adverse events remains and patients should have a clear understanding of the potential side effects of surgery. It requires clinicians to understand the risk and constantly improve the operation technologies to minimize the risk, and actively take corresponding countermeasures when adverse events occur.

Our study had some limitations. The SSDR funnel plot showed some asymmetry, indicating that publication bias is a non‐negligible limitation of this study. If present, it influences the results of the current meta‐analysis (especially for SSDR). In addition, because robot‐assisted surgery has not yet been widely accepted, we excluded studies involving robot‐assisted surgery. The results of our study showed that when there is satisfactory bilateral lymph node mapping, using a tracer with a high detection rate for the assessment of lymph node metastasis in patients with relatively small tumors is safe and reliable. ICG seems to be a superior SLN mapping technique compared with other methods. The weakness of this study is that it is not possible to explain the risk of abandoning systematic lymphadenectomy in the case of negative SLNs, especially in terms of long‐term tumor safety due to the lack of prospective evidence. Some other issues remain controversial such as the low accuracy of intraoperative SLN status evaluation by frozen section and the impact of micrometastasis on the prognosis. Larger‐scale, multi‐center studies are required in the future to further ensure safety.

## CONFLICT OF INTEREST

The authors declare that they have no conflicts of interest.

## AUTHORS’ CONTRIBUTIONS

All authors contributed to the conception and design of this review. Literature search and selection were performed by Wang Sixue and Yi Mingyu. Data extraction and quality evaluation were performed by Bao Bingting and Zhang Xinyue. The data analysis was performed by Zhang Xinyue and Jiang Li. The first draft of the manuscript was written by Zhang Xinyue and Bao Bingting, and all authors commented on previous versions of the manuscript. All authors read and approved the final manuscript.

## FUNDING INFORMATION

This work was supported by the National Natural Science Foundation of China under project number 81671437, as well as the Natural Science Foundation of Hunan Province under project number 2016JC2049.

## Data Availability

All data supporting this meta‐analysis are from previously reported studies and datasets, which have been cited. The processed data are available from the corresponding author upon request.
